# Edge universality for non-Hermitian random matrices

**DOI:** 10.1007/s00440-020-01003-7

**Published:** 2020-09-25

**Authors:** Giorgio Cipolloni, László Erdős, Dominik Schröder

**Affiliations:** 1 IST Austria, Am Campus 1, 3400 Klosterneuburg, Austria; 2 Institute for Theoretical Studies, ETH Zurich, Clausiusstr. 47, 8092 Zurich, Switzerland

**Keywords:** Ginibre ensemble, Universality, Circular law, Girko’s formula, 60B20, 15B52

## Abstract

We consider large non-Hermitian real or complex random matrices $X$ with independent, identically distributed centred entries. We prove that their local eigenvalue statistics near the spectral edge, the unit circle, coincide with those of the Ginibre ensemble, i.e. when the matrix elements of $X$ are Gaussian. This result is the non-Hermitian counterpart of the universality of the Tracy–Widom distribution at the spectral edges of the Wigner ensemble.

## Introduction

Following Wigner’s motivation from physics, most universality results on the local eigenvalue statistics for large random matrices concern the Hermitian case. In particular, the celebrated Wigner–Dyson statistics in the bulk spectrum [44], the Tracy–Widom statistics [56, 57] at the spectral edge and the Pearcey statistics [47, 58] at the possible cusps of the eigenvalue density profile all describe eigenvalue statistics of a large Hermitian random matrix. In the last decade there has been a spectacular progress in verifying Wigner’s original vision, formalized as the Wigner–Dyson–Mehta conjecture, for Hermitian ensembles with increasing generality, see e.g. [2, 15, 23–26, 35, 37, 40, 42, 45, 48, 52, 52] for the bulk, [5, 12, 13, 34, 38, 39, 46, 50, 53] for the edge and more recently [17, 22, 33] at the cusps.

Much less is known about the spectral universality for non-Hermitian models. In the simplest case of the Ginibre ensemble, i.e. random matrices with i.i.d. standard Gaussian entries without any symmetry condition, explicit formulas for all correlation functions have been computed first for the complex case [31] and later for the more complicated real case [10, 36, 49] (with special cases solved earlier [20, 21, 43]). Beyond the explicitly computable Ginibre case only the method of *four moment matching* by Tao and Vu has been available. Their main universality result in [54] states that the local correlation functions of the eigenvalues of a random matrix $X$ with i.i.d. matrix elements coincide with those of the Ginibre ensemble as long as the first four moments of the common distribution of the entries of $X$ (almost) match the first four moments of the standard Gaussian. This result holds for both real and complex cases as well as throughout the spectrum, including the edge regime.

In the current paper we prove the edge universality for any $n\times n$ random matrix $X$ with centred i.i.d. entries in the edge regime, in particular we remove the four moment matching condition from [54]. More precisely, under the normalization ${{\,\mathrm{\mathbf {E}}\,}}\left|x_{ab}\right|^2= \frac{1}{n}$, the spectrum of $X$ converges to the unit disc with a uniform spectral density according to the *circular law* [6–8, 30, 32, 51]. The typical distance between nearest eigenvalues is of order $n^{-1/2}$. We pick a reference point $z$ on the boundary of the limiting spectrum, $\left|z\right|=1$, and rescale correlation functions by a factor of $n^{-1/2}$ to detect the correlation of individual eigenvalues. We show that these rescaled correlation functions converge to those of the Ginibre ensemble as $n\rightarrow \infty$. This result is the non-Hermitian analogue of the Tracy–Widom edge universality in the Hermitian case. A similar result is expected to hold in the bulk regime, i.e. for any reference point $\left|z\right|<1$, but our method is currently restricted to the edge.

Investigating spectral statistics of non-Hermitian random matrices is considerably more challenging than Hermitian ones. We give two fundamental reasons for this: the first one is already present in the proof of the circular law on the global scale. The second one is specific to the most powerful existing method to prove universality of eigenvalue fluctuations.

The first issue a general one; it is well known that non-Hermitian, especially non-normal spectral analysis is difficult because, unlike in the Hermitian case, the resolvent $(X-z)^{-1}$ of a non-normal matrix is not effective to study eigenvalues near $z$. Indeed, $(X-z)^{-1}$ can be very large even if $z$ is away from the spectrum, a fact that is closely related to the instability of the non-Hermitian eigenvalues under perturbations. The only useful expression to grasp non-Hermitian eigenvalues is Girko’s celebrated formula, see (14) later, expressing linear statistics of eigenvalues of $X$ in terms of the log-determinant of the symmetrized matrix

1 $$\begin{aligned} H^z= \left( \begin{matrix} 0 &{} X-z \\ X^*-\overline{z} &{} 0 \end{matrix} \right) . \end{aligned}$$

Girko’s formula is much more subtle and harder to analyse than the analogous expression for the Hermitian case involving the boundary value of the resolvent on the real line. In particular, it requires a good lower bound on the smallest singular value of $X-z$, a notorious difficulty behind the proof of the circular law. Furthermore, any conceivable universality proof would rely on a local version of the circular law as an a priori control. Local laws on optimal scale assert that the eigenvalue density on a scale $n^{-1/2+\epsilon }$ is deterministic with high probability, i.e. it is a law of large number type result and is not sufficiently refined to detect correlations of individual eigenvalues. The proof of the local circular law requires a careful analysis of $H^z$ that has an additional structural instability due to its block symmetry. A specific estimate, tailored to Girko’s formula, on the trace of the resolvent of $(H^z)^2$ was the main ingredient behind the proof of the local circular law on optimal scale [14, 16, 59], see also [54] under three moment matching condition. Very recently the optimal local circular law was even proven for ensembles with inhomogeneous variance profiles in the bulk [3] and at the edge [4], the latter result also gives an optimal control on the spectral radius. An optimal local law for $H^z$ in the edge regime previously had not been available, even in the i.i.d. case.

The second major obstacle to prove universality of fluctuations of non-Hermitian eigenvalues is the lack of a good analogue of the Dyson Brownian motion. The essential ingredient behind the strongest universality results in the Hermitian case is the Dyson Brownian motion (DBM) [19], a system of coupled stochastic differential equations (SDE) that the eigenvalues of a natural stochastic flow of random matrices satisfy, see [27] for a pedagogical summary. The corresponding SDE in the non-Hermitian case involves not only eigenvalues but overlaps of eigenvectors as well, see e.g. [11, Appendix A]. Since overlaps themselves have strong correlation whose proofs are highly nontrivial even in the Ginibre case [11, 29], the analysis of this SDE is currently beyond reach.

Our proof of the edge universality circumvents DBM and it has two key ingredients. The first main input is an optimal local law for the resolvent of $H^z$ both in *isotropic* and *averaged* sense, see (13) later, that allows for a concise and transparent comparison of the joint distribution of several resolvents of $H^z$ with their Gaussian counterparts by following their evolution under the natural Ornstein-Uhlenbeck (OU). We are able to control this flow for a long time, similarly to an earlier proof of the Tracy–Widom law at the spectral edge of a Hermitian ensemble [41]. Note that the density of eigenvalues of $H^z$ develops a cusp as $\left|z\right|$ passes through 1, the spectral radius of $X$. The optimal local law for very general Hermitian ensembles in the cusp regime has recently been proven [22], strengthening the non-optimal result in [2]. This optimality was essential in the proof of the universality of the Pearcey statistics for both the complex Hermitian [22] and real symmetric [17] matrices with a cusp in their density of states. The matrix $H^z$, however, does not satisfy the key *flatness* condition required [22] due its large zero blocks. A very delicate analysis of the underlying matrix Dyson equation was necessary to overcome the flatness condition and prove the optimal local law for $H^z$ in [3, 4].

Our second key input is a lower tail estimate on the lowest singular value of $X-z$ when $\left|z\right|\approx 1$. A very mild regularity assumption on the distribution of the matrix elements of $X$, see (4) later, guarantees that there is no singular value below $n^{-100}$, say. Cruder bounds guarantee that there cannot be more than $n^\epsilon$ singular values below $n^{-3/4}$; note that this natural scaling reflects the cusp at zero in the density of states of $H^z$. Such information on the possible singular values in the regime $[n^{-100}, n^{-3/4}]$ is sufficient for the optimal local law since it is insensitive to $n^\epsilon$-eigenvalues, but for universality every eigenvalue must be accounted for. We therefore need a stronger lower tail bound on the lowest eigenvalue $\lambda _1$ of $(X-z)(X-z)^*$. With supersymmetric methods we recently proved [18] a precise bound of the form

2 $$\begin{aligned} {{\,\mathrm{\mathbf {P}}\,}}\Bigl (\lambda _1 \big ( (X-z)(X-z)^*\big ) \le \frac{x}{n^{3 /2}}\Bigr )\lesssim {\left\{ \begin{array}{ll} x+ \sqrt{x} e^{- n(\mathfrak {I}z)^2},&{} X\sim \text {Gin}(\mathbb {R})\\ x,&{} X\sim \text {Gin}(\mathbb {C}), \end{array}\right. } \end{aligned}$$

modulo logarithmic corrections, for the Ginibre ensemble whenever $\left|z\right|=1 + \mathcal {O}(n^{-1/2})$. Most importantly, (2) controls $\lambda _1$ on the optimal $n^{-3/2}$ scale and thus excluding singular values in the intermediate regime $[n^{-100}, n^{-3/4-\epsilon }]$ that was inaccessible with other methods. We extend this control to $X$ with i.i.d. entries from the Ginibre ensemble with Green function comparison argument using again the optimal local law for $H^z$.

### Notations and conventions

We introduce some notations we use throughout the paper. We write $\mathbb {H}$ for the upper half-plane $\mathbb {H}:=\{z\in \mathbb {C}\vert \mathfrak {I}z>0\}$, and for any $z\in \mathbb {C}$ we use the notation ${\text {d}}\!{}z:= 2^{-1} \text {i}({\text {d}}\!{}z\wedge {\text {d}}\!{}\overline{z})$ for the two dimensional volume form on $\mathbb {C}$. For any $2n\times 2n$ matrix $A$ we use the notation $\langle A\rangle := (2n)^{-1}{{\,\mathrm{Tr}\,}}A$ to denote the normalized trace of $A$. For positive quantities $f,g$ we write $f\lesssim g$ and $f\sim g$ if $f \le C g$ or $c g\le f\le Cg$, respectively, for some constants $c,C>0$ which depends only on the constants appearing in (3). We denote vectors by bold-faced lower case Roman letters ${\varvec{x}}, {\varvec{y}}\in \mathbb {C}^k$, for some $k\in \mathbb {N}$. Vector and matrix norms, $\Vert \varvec{x}\Vert$ and $\Vert A\Vert$, indicate the usual Euclidean norm and the corresponding induced matrix norm. Moreover, for a vector ${\varvec{x}}\in \mathbb {C}^k$, we use the notation ${\text {d}}\!{}{\varvec{x}}:= {\text {d}}\!{}x_1\dots {\text {d}}\!{}x_k$.

We will use the concept of “with very high probability” meaning that for any fixed $D>0$ the probability of the event is bigger than $1-n^{-D}$ if $n\ge n_0(D)$. Moreover, we use the convention that $\xi >0$ denotes an arbitrary small constant.

We use the convention that quantities without tilde refer to a general matrix with i.i.d. entries, whilst any quantity with tilde refers to the Ginibre ensemble, e.g. we use $X$, $\{\sigma _i\}_{i=1}^n$ to denote a non-Hermitian matrix with i.i.d. entries and its eigenvalues, respectively, and $\widetilde{X}$, $\{\widetilde{\sigma }_i\}_{i=1}^n$ to denote their Ginibre counterparts.

## Model and main results

We consider real or complex i.i.d. matrices $X$, i.e. matrices whose entries are independent and identically distributed as $x_{ab} {\mathop {=}\limits ^{d}} n^{-1/2}\chi$ for a random variable $\chi$. We formulate two assumptions on the random variable $\chi$:

### Assumption (A)

In the real case we assume that ${{\,\mathrm{\mathbf {E}}\,}}\chi =0$ and ${{\,\mathrm{\mathbf {E}}\,}}\chi ^2=1$, while in the complex case we assume ${{\,\mathrm{\mathbf {E}}\,}}\chi ={{\,\mathrm{\mathbf {E}}\,}}\chi ^2=0$ and ${{\,\mathrm{\mathbf {E}}\,}}\left|\chi \right|^2=1$. In addition, we assume the existence of high moments, i.e. that there exist constants $C_p>0$ for each $p\in \mathbb {N}$, such that

3 $$\begin{aligned} {{\,\mathrm{\mathbf {E}}\,}}\left|\chi \right|^p\le C_p. \end{aligned}$$

### Assumption (B)

There exist $\alpha ,\beta >0$ such that the probability density $g:\mathbb {F}\rightarrow [0,\infty )$ of the random variable $\chi$ satisfies

4 $$\begin{aligned} g \in L^{1+\alpha }(\mathbb {F}),\qquad \Vert g\Vert _{1+\alpha }\le n^\beta , \end{aligned}$$

where $\mathbb {F}=\mathbb {R},\mathbb {C}$ in the real and complex case, respectively.

### Remark 1

We remark that we use Assumption (B) only to control the probability of a very small singular value of $X-z$. Alternatively, one may use the statement

5 $$\begin{aligned} {{\,\mathrm{\mathbf {P}}\,}}( {{\,\mathrm{Spec}\,}}(H^z) \cap [-n^{-l}, n^{-l}] =\emptyset )\le C_l n^{-l/2}, \end{aligned}$$

for any $l\ge 1$, uniformly in $\left|z\right|\le 2$, that follows directly from [55, Theorem 3.2] without Assumption (B). Using (5) makes Assumption (B) superfluous in the entire paper, albeit at the expense of a quite sophisticated proof.

We denote the eigenvalues of $X$ by $\sigma _1,\dots ,\sigma _n\in \mathbb {C}$, and define the $k$-*point correlation function* $p_k^{(n)}$ of $X$ implicitly such that

6 $$\begin{aligned}&\int _{\mathbb {C}^k}F(z_1,\dots , z_k) p_k^{(n)}(z_1,\dots , z_k)\, {\text {d}}\!{}z_1\dots {\text {d}}\!{}z_k\nonumber \\&\qquad =\left( {\begin{array}{c}n\\ k\end{array}}\right) ^{-1}{{\,\mathrm{\mathbf {E}}\,}}\sum _{i_1,\dots , i_k} F(\sigma _{i_1}, \dots , \sigma _{i_k}), \end{aligned}$$

for any smooth compactly supported test function $F:\mathbb {C}^k \rightarrow \mathbb {C}$, with $i_j\in \{1,\dots , n\}$ for $j\in \{1,\dots ,k\}$ all distinct. For the important special case when $\chi$ follows a standard real or complex Gaussian distribution, we denote the $k$-point function of the *Ginibre matrix* $X$ by $p_k^{(n,\text {Gin}(\mathbb {F}))}$ for $\mathbb {F}=\mathbb {R},\mathbb {C}$. The *circular law* implies that the $1$-point function converges

$$\begin{aligned} \lim _{n\rightarrow \infty }p_1^{(n)}(z) = \frac{1}{\pi } \mathbf {1}(z\in \mathbb {D}) = \frac{1}{\pi }\mathbf {1}(\left|z\right|\le 1) \end{aligned}$$

to the uniform distribution on the unit disk. On the scale $n^{-1/2}$ of individual eigenvalues the scaling limit of the $k$-point function has been explicitly computed in the case of complex and real Ginibre matrices, $X\sim \text {Gin}(\mathbb {R}),\text {Gin}(\mathbb {C})$, i.e. for any fixed $z_1,\dots ,z_k,w_1,\dots ,w_k\in \mathbb {C}$ there exist scaling limits $p_{z_1,\dots ,z_k}^{(\infty )}=p_{z_1,\dots ,z_k}^{(\infty ,\text {Gin}(\mathbb {F}))}$ for $\mathbb {F}=\mathbb {R},\mathbb {C}$ such that

7 $$\begin{aligned} \lim _{n\rightarrow \infty } p_k^{(n,\text {Gin}(\mathbb {F}))}\Bigl (z_1+\frac{w_1}{n^{1/2}},\dots ,z_k+\frac{w_k}{n^{1/2}}\Bigr ) = p_{z_1,\dots ,z_k}^{(\infty ,\text {Gin}(\mathbb {F}))}(w_1,\dots ,w_k). \end{aligned}$$

### Remark 2

The $k$-point correlation function $p_{z_1,\dots , z_k}^{(\infty ,\text {Gin}(\mathbb {F}))}$ of the Ginibre ensemble in both the complex and real cases $\mathbb {F}=\mathbb {C},\mathbb {R}$ is explicitly known; see [31] and [44] for the complex case, and [10, 20, 28] for the real case, where the appearance of $\sim n^{1/2}$ real eigenvalues causes a singularity in the density. In the complex case $p_{z_1,\dots , z_k}^{(\infty ,\text {Gin}(\mathbb {C}))}$ is determinantal, i.e. for any $w_1,\dots , w_k\in \mathbb {C}$ it holds

$$\begin{aligned} p_{z_1,\dots , z_k}^{(\infty ,\text {Gin}(\mathbb {C}))}(w_1,\dots ,w_k)=\det \left( K_{z_i,z_j}^{(\infty ,\text {Gin}(\mathbb {C}))}(w_i,w_j)\right) _{1\le i,j\le k} \end{aligned}$$

where for any complex numbers $z_1$, $z_2$, $w_1$, $w_2$ the kernel $K_{z_1,z_2}^{(\infty ,\text {Gin}(\mathbb {C}))}(w_1,w_2)$ is defined by

(i) For $$z_1\ne z_2$$, $$K_{z_1,z_2}^{(\infty ,\text {Gin}(\mathbb {C}))}(w_1,w_2)=0$$.
(ii) For $z_1=z_2$ and $\left|z_1\right|>1$, $K_{z_1,z_2}^{(\infty ,\text {Gin}(\mathbb {C}))}(w_1,w_2)=0$.
(iii) For $z_1=z_2$ and $\left|z_1\right|<1$,
  $$\begin{aligned} K_{z_1,z_2}^{(\infty ,\text {Gin}(\mathbb {C}))}(w_1,w_2)=\frac{1}{\pi }e^{-\frac{\left|w_1\right|^2}{2} -\frac{\left|w_2\right|^2}{2}+w_1\overline{w_2}}. \end{aligned}$$
(iv) For $z_1=z_2$ and $\left|z_1\right|=1$,
  $$\begin{aligned} K_{z_1,z_2}^{(\infty ,\text {Gin}(\mathbb {C}))}(w_1,w_2)=\frac{1}{2\pi }\left[ 1+ {{\,\mathrm{erf}\,}}\left( -\sqrt{2}(z_1\overline{w_2}+w_1\overline{z_2})\right) \right] e^{-\frac{\left|w_1\right|^2}{2}-\frac{\left|w_2\right|^2}{2}+w_1\overline{w_2}}, \end{aligned}$$
  where
  $$\begin{aligned} {{\,\mathrm{erf}\,}}(z):= \frac{2}{\sqrt{\pi }}\int _{\gamma _z} e^{-t^2}\, {\text {d}}\!{}t, \end{aligned}$$
  for any $z\in \mathbb {C}$, with $\gamma _z$ any contour from $0$ to $z$.

For the corresponding much more involved formulas for $p_k^{(\infty ,\text {Gin}(\mathbb {R}))}$ we refer the reader to [10].

Our main result is the universality of $p_{z_1,\dots ,z_k}^{(\infty ,\text {Gin}(\mathbb {R},\mathbb {C}))}$ at the edge. In particular we show, that the edge-scaling limit of $p_k^{(n)}$ agrees with the known scaling limit of the corresponding real or complex Ginibre ensemble.

### Theorem 1

(Edge universality) Let $X$ be an i.i.d. $n\times n$ matrix, whose entries satisfy Assumption (A) and (B). Then, for any fixed integer $k\ge 1$, and complex spectral parameters $z_1, \dots , z_k$ such that $\left|z_j\right|^2=1$, $j=1,\dots ,k$, and for any compactly supported smooth function $F:\mathbb {C}^k \rightarrow \mathbb {C}$, we have the bound

8 $$\begin{aligned} \int _{\mathbb {C}^k} F(\mathbf{w})\left[ p_k^{(n)} \left( \mathbf{z}+\frac{\mathbf{w}}{\sqrt{n}}\right) -p_{\mathbf{z}}^{(\infty ,\text {Gin}(\mathbb {F}))}(\mathbf{w}) \right] \, {\text {d}}\!{}\mathbf{w}=\mathcal {O}( n^{-c}), \end{aligned}$$

where the constant in $\mathcal {O}(\cdot )$ may depend on $k$ and the $C^{2k+1}$ norm of $F$, and $c>0$ is a small constant depending on $k$.

### Proof strategy

For the proof of Theorem 1 it is essential to study the linearized $2n\times 2n$ matrix $H^z$ defined in (1) with eigenvalues $\lambda _1^z\le \dots \le \lambda _{2n}^z$ and resolvent $G(w)=G^z(w):= (H^z-w)^{-1}$. We note that the block structure of $H^z$ induces a spectrum symmetric around $0$, i.e. $\lambda _i^z=-\lambda _{2n-i+1}^z$ for $i=1,\dots ,n$. The resolvent becomes approximately deterministic as $n\rightarrow \infty$ and its limit can be found by solving the simple scalar equation

9 $$\begin{aligned} -\frac{1}{\widehat{m}^z}= w + \widehat{m}^z- \frac{\left|z\right|^2}{w+\widehat{m}^z},\quad \widehat{m}^z(w)\in \mathbb {H},\quad w\in \mathbb {H}, \end{aligned}$$

which is a special case of the *matrix Dyson equation (MDE)*, see e.g. [1]. In the following we may often omit the $z$-dependence of $\widehat{m}^z$, $G^z(w)$, $\dots$, in the notation. We note that on the imaginary axis we have $\widehat{m}(\text {i}\eta )=\text {i}\mathfrak {I}\widehat{m}(\text {i}\eta )$, and in the edge regime $\left|1-\left|z\right|^2\right|\lesssim n^{-1/2}$ we have the scaling [4, Lemma 3.3]

10 $$\begin{aligned} \mathfrak {I}\widehat{m}(\text {i}\eta ) \sim \left. {\left\{ \begin{array}{ll} \left|1-\left|z\right|^2\right|^{1/2} + \eta ^{1/3}, &{} \left|z\right|\le 1,\\ \frac{\eta }{\left|1-\left|z\right|^2\right|+\eta ^{2/3}}, &{} \left|z\right|>1 \end{array}\right. }\right\} \lesssim n^{-1/4}+\eta ^{1/3}. \end{aligned}$$

For $\eta >0$ we define

11 $$\begin{aligned} u=u^z(\text {i}\eta ):= \frac{\mathfrak {I}\widehat{m}(\text {i}\eta )}{\eta + \mathfrak {I}\widehat{m}(\text {i}\eta )},\qquad M=M^z(\text {i}\eta ):= \begin{pmatrix}\widehat{m}(\text {i}\eta )&{} -z u(\text {i}\eta )\\ -\overline{z} u(\text {i}\eta ) &{} \widehat{m}(\text {i}\eta ) \end{pmatrix}, \end{aligned}$$

where $M$ should be understood as a $2n\times 2n$ whose four $n\times n$ blocks are all multiples of the identity matrix, and we note that [4, Eq. (3.62)]

12 $$\begin{aligned} u(\text {i}\eta )\lesssim 1,\quad \Vert M(\text {i}\eta )\Vert \lesssim 1,\quad \Vert M'(\text {i}\eta )\Vert \lesssim \frac{1}{\eta ^{2/3}} \end{aligned}$$

Throughout the proof we shall make use of the following optimal local law which is a direct consequence of [4, Theorem 5.2] (extending [3, Theorem 5.2] to the edge regime). Compared to [4] we require the local law simultaneously in all the spectral parameters $z,\eta$ and for $\eta$ slightly below the fluctuation scale $n^{-3/4}$. We defer the proofs for both extensions to “Appendix A”.

#### Proposition 1

(Local law for $H^z$) Let $X$ be an i.i.d. $n\times n$ matrix, whose entries satisfy Assumption (A) and (B), and let $H^z$ be as in (1). Then for any deterministic vectors $\varvec{x},\varvec{y}$ and matrix $R$ and any $\xi >0$ the following holds true with very high probability: Simultaneously for any $z$ with for $\left|1-\left|z\right|\right|\lesssim n^{-1/2}$ and all $\eta$ such that $n^{-1}\le \eta \le n^{100}$ we have the bounds

13 $$\begin{aligned} \left|\langle \varvec{x},(G^z(\text {i}\eta )-M^z(\text {i}\eta ))\varvec{y}\rangle \right|&\le n^\xi \Vert \varvec{x}\Vert \Vert \varvec{y}\Vert \Bigl (\frac{1}{n^{1/2}\eta ^{1/3}}+\frac{1}{n\eta }\Bigr ), \nonumber \\ \left|\langle R(G^z(\text {i}\eta )-M^z(\text {i}\eta ))\rangle \right|&\le \frac{n^\xi \Vert R\Vert }{n\eta }. \end{aligned}$$

For the application of Proposition 1 towards the proof of Theorem 1 the special case of $R$ being the identity matrix, and $\varvec{x},\varvec{y}$ being either the standard basis vectors, or the vectors $\varvec{1}_\pm$ of zeros and ones defined later in (58).

The linearized matrix $H^z$ can be related to the eigenvalues $\sigma _i$ of $X$ via Girko’s Hermitization formula [32, 54]

14 $$\begin{aligned} \frac{1}{n} \sum _i f_{z_0}(\sigma _i)&= \frac{1}{4\pi n}\int _\mathbb {C}\varDelta f_{z_0}(z)\log \left|\det H_z\right|{\text {d}}\!{}z\nonumber \\&= -\frac{1}{4\pi n}\int _\mathbb {C}\varDelta f_{z_0}(z)\int _0^\infty \mathfrak {I}{{\,\mathrm{Tr}\,}}G^z(\text {i}\eta ){\text {d}}\!{}\eta {\text {d}}\!{}z \end{aligned}$$

for rescaled test functions $f_{z_0}(z):= n f(\sqrt{n}(z-z_0))$, where $f:\mathbb {C}\rightarrow \mathbb {C}$ is smooth and compactly supported. When using (14) the small $\eta$ regime requires additional bounds on the number of small eigenvalues $\lambda _i^z$ of $H^z$, or equivalently small singular values of $X-z$. For very small $\eta$, say $\eta \le n^{-100}$, the absence of eigenvalues below $\eta$, can easily be ensured by Assumption (B). For $\eta$ just below the critical scale of $n^{-3/4}$, however, we need to prove an additional bound on the number of eigenvalues, as stated below.

#### Proposition 2

For any $n^{-1}\le \eta \le n^{-3/4}$ and $\left|\left|z\right|^2-1\right|\lesssim n^{-1/2}$ we have the bound

15 $$\begin{aligned} {{\,\mathrm{\mathbf {E}}\,}}\left|\{i \vert \left|\lambda _i^z\right|\le \eta \}\right|&\lesssim {\left\{ \begin{array}{ll} n^{3/2} \eta ^2 (1+\left|\log (n\eta ^{4/3})\right|), &{}\text {X complex} \\ n^{3/4} \eta , &{}\text {X real} \end{array}\right. }\nonumber \\&\qquad +\mathcal {O}(\frac{n^\xi }{n^{5/2} \eta ^3}), \end{aligned}$$

on the number of small eigenvalues, for any $\xi >0$.

We remark that the precise asymptotics of (15) are of no importance for the proof of Theorem 1. Instead it would be sufficient to establish that for any $\epsilon >0$ there exists $\delta >0$ such that we have ${{\,\mathrm{\mathbf {E}}\,}}\left|\{i \vert \left|\lambda _i^z\right|\le n^{-3/4-\epsilon }\}\right|\lesssim n^{-\delta }$.

The paper is organized as follows: in Sect. 3 we will prove Proposition 2 by a Green function comparison argument, using the analogous bound for the Gaussian case, as recently obtained in [18]. In Sect. 4 we will then present the proof of our main result, Theorem 1, which follows from combining the local law (13), Girko’s Hermitization identity (14), the bound on small singular values (15) and another long-time Green function comparison argument.

## Estimate on the lower tail of the smallest singular value of $X-z$

The main result of this section is an estimate of the lower tail of the density of the smallest $\left|\lambda _i^z\right|$ in Proposition 2. For this purpose we introduce the following flow

16 $$\begin{aligned} {\text {d}}\!{}X_t=-\frac{1}{2} X_t {\text {d}}\!{}t+\frac{{\text {d}}\!{}B_t}{\sqrt{n}}, \end{aligned}$$

with initial data $X_0=X$, where $B_t$ is the real or complex matrix valued standard Brownian motion, i.e. $B_t\in \mathbb {R}^{n\times n}$ or $B_t\in \mathbb {C}^{n\times n}$, accordingly with $X$ being real or complex, where $(b_t)_{ab}$ in the real case, and $\sqrt{2}\mathfrak {R}[(b_t)_{ab}], \sqrt{2}\mathfrak {I}[(b_t)_{ab}]$ in the complex case, are independent standard real Brownian motions for $a,b\in [n]$. The flow (16) induces a flow ${\text {d}}\!{}\chi _t=-\chi _t{\text {d}}\!{}t/2+{\text {d}}\!{}b_t$ on the entry distribution $\chi$ with solution

17 $$\begin{aligned} \chi _t = e^{-t/2}\chi + \int _0^t e^{-(t-s)/2}{\text {d}}\!{}b_s,\quad \text {i.e.}\quad \chi _t{\mathop {=}\limits ^{d}}e^{-t/2}\chi + \sqrt{1-e^{-t}}g, \end{aligned}$$

where $g {\sim } {\mathcal {N}}(0,1)$ is a standard real or complex Gaussian, independent of $\chi$, with ${{\,\mathrm{\mathbf {E}}\,}}g^2=0$ in the complex case. By linearity of cumulants we find

18 $$\begin{aligned} \kappa _{i,j}(\chi _t)=e^{-(i+j)t/2}\kappa _{i,j}(\chi ) + {\left\{ \begin{array}{ll} (1-e^{-t}) \kappa _{i,j}(g), &{} i+j=2\\ 0, &{}\text {else}, \end{array}\right. } \end{aligned}$$

where $\kappa _{i,j}(x)$ denotes the joint cumulant of $i$ copies of $x$ and $j$ copies of $\overline{x}$, in particular $\kappa _{2,0}(x)=\kappa _{0,2}(x)=\kappa _{1,1}(x)=1$ for $x=\chi ,g$ in the real case, and $\kappa _{0,2}(x)=\kappa _{2,0}(x)=0\ne \kappa _{1,1}(x)=1$ for $x=\chi ,g$ in the complex case.

Thus (17) implies that, in distribution,

19 $$\begin{aligned} X_t{\mathop {=}\limits ^{d}} e^{-t/2} X_0+\sqrt{1-e^{-t}} \widetilde{X}, \end{aligned}$$

where $\widetilde{X}$ is a real or complex Ginibre matrix independent of $X_0=X$. Then, we define the $2n\times 2n$ matrix $H_t=H_t^z$ as in (1) replacing $X$ by $X_t$, and its resolvent $G_t(w)=G_t^z(w):= (H_t-w)^{-1}$, for any $w\in \mathbb {H}$. We remark that we defined the flow in (16) with initial data $X$ and not $H^z$ in order to preserve the shape of the self consistent density of states of the matrix $H_t$ along the flow. In particular, by (16) it follows that $H_t$ is the solution of the flow

20 $$\begin{aligned} {\text {d}}\!{}H_t=-\frac{1}{2}(H_t+Z){\text {d}}\!{}t+\frac{{\text {d}}\!{}\mathfrak {B}_t}{\sqrt{n}},\quad H_0=H=H^z \end{aligned}$$

with

$$\begin{aligned} Z:= \left( \begin{matrix} 0 &{} zI \\ \overline{z}I &{} 0 \end{matrix}\right) , \qquad \mathfrak {B}_t:= \left( \begin{matrix} 0 &{} B_t \\ B_t^* &{} 0 \end{matrix}\right) , \end{aligned}$$

where $I$ denotes the $n \times n$ identity matrix.

### Proposition 3

Let $R_t:= \langle G_t(\text {i}\eta ) \rangle =\text {i}\langle \mathfrak {I}G_t(\text {i}\eta )\rangle$, then for any $n^{-1}\le \eta \le n^{-3/4}$ it holds that

21 $$\begin{aligned} \left| {{\,\mathrm{\mathbf {E}}\,}}[R_{t_2}-R_{t_1}] \right| \lesssim \frac{(e^{-3t_1/2}-e^{-3t_2/2}) n^\xi }{n^{7/2}\eta ^4}, \end{aligned}$$

for any arbitrary small $\xi >0$ and any $0\le t_1< t_2\le +\infty$, with the convention that $e^{-\infty }=0$.

### Proof

Denote $W_t:=H_t+Z$. By (20) and Ito’s Lemma it follows that

22 $$\begin{aligned} {{\,\mathrm{\mathbf {E}}\,}}\frac{{\text {d}}\!{}R_t}{{\text {d}}\!{}t}={{\,\mathrm{\mathbf {E}}\,}}\left[ -\frac{1}{2}\sum _\alpha w_\alpha (t) \partial _\alpha R_t+\frac{1}{2}\sum _{\alpha ,\beta }\kappa _t(\alpha ,\beta ) \partial _\alpha \partial _\beta R_t\right] , \end{aligned}$$

where $\alpha , \beta \in [2n]^2$ are double indices, $w_\alpha (t)$ are the entries of $W_t$ and

23 $$\begin{aligned} \kappa _t(\alpha ,\beta ,,\dots ):=\kappa (w_\alpha (t), w_\beta (t),\dots ) \end{aligned}$$

denotes the joint cumulant of $w_\alpha ,w_\beta ,\dots$, and $\partial _\alpha := \partial _{w_\alpha }$. By (18) and the independence of $\chi$ and $g$ it follows that $\kappa _t(\alpha ,\beta )=\kappa _0(\alpha ,\beta )$ for all $\alpha ,\beta$ and

24 $$\begin{aligned}&\kappa _t(\alpha , \beta _1, \dots , \beta _j)\nonumber \\&={\left\{ \begin{array}{ll} e^{-t\frac{j+1}{2}} n^{-\frac{j+1}{2}} \kappa _{l,k}(\chi ) &{}\text {if }\alpha \not \in [n]^2\cup [n+1,2n]^2,\;\beta _i\in \{\alpha ,\alpha '\} \, \forall i\in [j]\\ 0 &{}\text {otherwise}, \end{array}\right. } \end{aligned}$$

for $j>1$, where for a double index $\alpha =(a,b)$, we use the notation $\alpha ':= (b,a)$, and $l,k$ with $l+k=j+1$ denote the number of double indices among $\alpha ,\beta _1,\dots ,\beta _j$ which correspond to the upper-right, or respectively lower-left corner of the matrix $H$. In the sequel the value of $\kappa _{k,l}(\chi )$ is of no importance, but we note that Assumption (A) ensures the bound $\left|\kappa _{k,l}(\chi )\right| \lesssim \sum _{j\le k+l} C_j<\infty$ for any $k,l$, with $C_j$ being the constants from Assumption (A).

We will use the cumulant expansion that holds for any smooth function $f$:

25 $$\begin{aligned} {{\,\mathrm{\mathbf {E}}\,}}w_\alpha f(w)= & {} \sum _{m=0}^{K} \sum _{\beta _1,\dots ,\beta _m\in [2n]^2} \frac{\kappa (\alpha ,\beta _1,\dots ,\beta _m)}{m!} {{\,\mathrm{\mathbf {E}}\,}}\partial _{\beta _1}\dots \partial _{\beta _m} f(w) \nonumber \\&+ \varOmega (K,f), \end{aligned}$$

where the error term $\varOmega (K,f)$ goes to zero as the expansion order $K$ goes to infinity. In our application the error is negligible for, say, $K=100$ since with each derivative we gain an additional factor of $n^{-1/2}$ and due to the independence (24) the sums of any order have effectively only $n^2$ terms. Applying (25) to (22) with $f=\partial _\alpha R_t$, the first order term is zero due to the assumption ${{\,\mathrm{\mathbf {E}}\,}}x_\alpha =0$, and the second order term cancels. The third order term is given by

26 $$\begin{aligned} \left| {\sum _{\alpha \beta _1\beta _2}\kappa _t(\alpha ,\beta _1,\beta _2){{\,\mathrm{\mathbf {E}}\,}}[\partial _\alpha \partial _{\beta _1}\partial _{\beta _2} R_t]}\right| \lesssim e^{-3t/2}\frac{n^\xi }{n^{7/2}\eta ^4}. \end{aligned}$$

### Proof of Eq. (26)

It follows from the resolvent identity that $\partial _\alpha G=-G\varDelta ^\alpha G$, where $\varDelta ^\alpha$ is the matrix of all zeros except for a $1$ in the $\alpha$-th entry.^1^ Thus, neglecting minuses and irrelevant constant factors, for any fixed $\alpha$, the sum (26) is given by a sum of terms of the form

$$\begin{aligned} \langle G_t \varDelta ^{\gamma _1} G_t\varDelta ^{\gamma _2} G_t\varDelta ^{\gamma _3} G_t \rangle , \qquad \gamma _1,\gamma _2,\gamma _3\in \{\alpha ,\alpha '\}. \end{aligned}$$

Hence, considering all possible choices of $\gamma _{1},\gamma _2,\gamma _3$ and using independence to conclude that $\kappa _t(\alpha ,\beta _1,\beta _2)$ can only be non-zero if $\beta _1,\beta _2\in \{\alpha ,\alpha '\}$ we arrive at

27 $$\begin{aligned}&\left| {\sum _{\alpha \beta _1\beta _2}\kappa _t(\alpha ,\beta _1,\beta _2) {{\,\mathrm{\mathbf {E}}\,}}[\partial _\alpha \partial _{\beta _1}\partial _{\beta _2} R_t]}\right| \nonumber \\&\lesssim e^{-3t/2} n^{-5/2} \biggl (\left| {\sum _{abc}\mathfrak {I}{{\,\mathrm{\mathbf {E}}\,}}G_{ca} G_{ba} G_{ba} G_{bc} }\right| +\left| {\sum _{abc}\mathfrak {I}{{\,\mathrm{\mathbf {E}}\,}}G_{ca} G_{ba} G_{bb} G_{ac} }\right| \nonumber \\&\qquad \qquad \qquad \qquad +\left| {\sum _{abc}\mathfrak {I}{{\,\mathrm{\mathbf {E}}\,}}G_{ca} G_{bb} G_{aa} G_{bc} }\right| \biggr ), \end{aligned}$$

where the sums are taken over $(a,b)\in [2n]^2\setminus ([n]^2\cup [n+1,2n]^2)$ and $c\in [2n]$, and we dropped the time dependence of $G=G_t$ for notational convenience.

We estimate the three sums in (27) using that, by (10), (12), it follows

$$\begin{aligned} \left|G_{ab}\right|\lesssim n^\xi ,\qquad \left|G_{aa}\right|\le \mathfrak {I}\widehat{m}+\left|(G-M)_{aa}\right|\lesssim n^{-1/4}+ \eta ^{1/3}+ \frac{n^\xi }{n\eta }\lesssim \frac{n^\xi }{n\eta }, \end{aligned}$$

from Proposition 1, and Cauchy-Schwarz estimates by

$$\begin{aligned} \sum _{abc} \left|G_{ca}G_{ba}G_{ba}G_{bc}\right|&\le \sum _{ab} \left|G_{ba}\right|^2 \sqrt{\sum _c \left|G_{ca}\right|^2}\sqrt{\sum _{c} \left|G_{bc}\right|^2 }\\&=\sum _{ab} \left|G_{ba}\right|^2 \sqrt{(G^*G)_{aa}}\sqrt{(GG^*)_{bb} } \\&= \frac{1}{\eta }\sum _{ab} \left|G_{ba}\right|^2 \sqrt{(\mathfrak {I}G)_{aa}}\sqrt{(\mathfrak {I}G)_{bb} } \lesssim \frac{n^{\xi }}{n\eta ^2} \sum _b (GG^*)_{bb} \\&= \frac{n^{\xi }}{n\eta ^3} \sum _b (\mathfrak {I}G)_{bb} \lesssim \frac{n^{2\xi }}{n\eta ^4}, \end{aligned}$$

and similarly

$$\begin{aligned} \sum _{abc} \left|G_{ca}G_{ba}G_{bb}G_{ac}\right|&\lesssim \frac{n^{\xi }}{n\eta ^2} \sum _{ab} \left|G_{ba}\right| (\mathfrak {I}G)_{aa} \\&\le \frac{n^{\xi }}{n^{1/2}\eta ^{5/2}} \sum _{a} (\mathfrak {I}G)_{aa} \sqrt{(\mathfrak {I}G)_{aa}} \lesssim \frac{n^{5\xi /2}}{n\eta ^4} \end{aligned}$$

and

$$\begin{aligned} \sum _{abc} \left|G_{ca}G_{bb}G_{aa}G_{bc}\right|&\lesssim \frac{n^{2\xi }}{n^2\eta ^3} \sum _{ab} \sqrt{(\mathfrak {I}G)_{aa}}\sqrt{(\mathfrak {I}G)_{bb}} \lesssim \frac{n^{3\xi }}{n\eta ^4}. \end{aligned}$$

This concludes the proof of (26) by choosing $\xi$ in Proposition 1 accordingly. $\square$

Finally, in the cumulant expansion of (22) we are able to bound the terms of order at least four trivially. Indeed, for the fourth order, the trivial bound is $e^{-2t}$ since the $n^3$ from the summation is compensated by the $n^{-2}$ from the cumulants and the $n^{-1}$ from the normalization of the trace. Morever, we can always perform at least two Ward-estimates on the first and last $G$ with respect to the trace index. Thus we can estimate any fourth-order term by $e^{-2t}(n\eta )^{-2}\le e^{-3t/2}n^{-7/2}\eta ^{-4}$, and we note that the power-counting for higher order terms is even better than that. Whence we have shown that ${{\,\mathrm{\mathbf {E}}\,}}\left|{\text {d}}\!{}R_t/{\text {d}}\!{}t\right| \lesssim e^{-3t/2}n^{-7/2}\eta ^{-4}$ and the proof of Proposition 3 is complete after integrating (22) in $t$ from $t_1$ to $t_2$. $\square$

Let $\widetilde{X}$ be a real or complex $n\times n$ Ginibre matrix and let $\widetilde{H}^z$ be the linearized matrix defined as in (1) replacing $X$ by $\widetilde{X}$. Let $\widetilde{\lambda }_i=\widetilde{\lambda }_i^z$, with $i\in \{1,\dots , 2n\}$, be the eigenvalues of $\widetilde{H}^z$. We define the non negative Hermitian matrix $\widetilde{Y}=\widetilde{Y}^z:= (\widetilde{X}-z)(\widetilde{X}-z)^*$, then, by [18],[Eq. (13c)-(14)] it follows that for any $\eta \le n^{-3/4}$ we have

28 $$\begin{aligned} {{\,\mathrm{\mathbf {E}}\,}}{{\,\mathrm{Tr}\,}}\big [\widetilde{Y}+\eta ^2\big ]^{-1}= {{\,\mathrm{\mathbf {E}}\,}}\sum _{i=1}^{2n} \frac{1}{\widetilde{\lambda }_i^2+\eta ^2}\lesssim {\left\{ \begin{array}{ll} n^{3/2} (1+\left|\log (n\eta ^{4/3})\right|), &{}\text {Gin}(\mathbb {C}), \\ n^{3/4} \eta ^{-1}, &{}\text {Gin}(\mathbb {R}), \end{array}\right. } \end{aligned}$$

for $\widetilde{X}$ distributed according to the complex, or respective, real Ginibre ensemble.

Combining (28) and Proposition 3 we now present the proof of Proposition 2.

### Proof of Proposition 2

Let $\lambda _i(t)$, with $i\in \{1,\dots , 2n\}$, be the eigenvalues of $H_t$ for any $t\ge 0$. Note that $\lambda _i(0)=\lambda _i$, since $H_0=H^z$. By (21), choosing $t_1=0$, $t_2=+\infty$ it follows that

29 $$\begin{aligned} {{\,\mathrm{\mathbf {E}}\,}}_{H_t} \left|\{i\vert \left|\lambda _i\right|\le \eta \}\right|&\le \eta \cdot {{\,\mathrm{\mathbf {E}}\,}}_{H_t} \left( \mathfrak {I}\sum _{i=1}^{2n} \frac{1}{\lambda _i-\text {i}\eta }\right) \nonumber \\&=\eta ^2 \cdot {{\,\mathrm{\mathbf {E}}\,}}_{H_\infty }\left( \sum _{i=1}^{2n} \frac{1}{\lambda _i^2+\eta ^2} \right) +{\mathcal {O}}\left( \frac{n^\xi }{n^{5/2} \eta ^3}\right) , \end{aligned}$$

for any $\xi >0$. Since the distribution of $H_\infty$ is the same as $\widetilde{H}^z$ it follows that

$$\begin{aligned} {{\,\mathrm{\mathbf {E}}\,}}_{\widetilde{H}^z}\left( \sum _{i=1}^{2n} \frac{1}{\mu _i^2+\eta ^2} \right) =2{{\,\mathrm{\mathbf {E}}\,}}_{\widetilde{X}} {{\,\mathrm{Tr}\,}}\big [\widetilde{Y}+\eta ^2\big ]^{-1}, \end{aligned}$$

and combining (28) with (29), we immediately conclude the bound in (15). $\square$

## Edge universality for non-Hermitian random matrices

In this section we prove our main edge universality result, as stated in Theorem 1. In the following of this section without loss of generality we can assume that the test function $F$ is of the form

30 $$\begin{aligned} F(w_1,\dots , w_k)=f^{(1)}(w_1)\cdots f^{(k)}(w_k), \end{aligned}$$

with $f^{(1)},\dots , f^{(k)}:\mathbb {C}\rightarrow \mathbb {C}$ being smooth and compactly supported functions. Indeed, any smooth function $F$ can be effectively approximated by its truncated Fourier series (multiplied by smooth cutoff function of product form); see also [54, Remark 3]. Using the effective decay of the Fourier coefficients of $F$ controlled by its $C^{2k+1}$ norm, a standard approximation argument shows that if (8) holds for $F$ in the product form (30) with an error $\mathcal {O}( n^{-c(k)})$, then it also holds for a general smooth function with an error $\mathcal {O}( n^{-c})$, where the implicit constant in $\mathcal {O}(\cdot )$ depends on $k$ and on the $C^{2k+1}$-norm of $F$, and the constant $c>0$ depends on $k$.

To resolve eigenvalues on their natural scale we consider the rescaling $f_{z_0}(z):= n f(\sqrt{n}(z-z_0))$ and compare the linear statistics $n^{-1}\sum _i f_{z_0}(\sigma _i)$ and $n^{-1}\sum _i f_{z_0}(\widetilde{\sigma }_i)$, with $\sigma _i,\widetilde{\sigma }_i$ being the eigenvalues of $X$ and of the comparison Ginibre ensemble $\widetilde{X}$, respectively. For convenience we may normalize both linear statistics by their deterministic approximation from the local law (13) which, according to (14) is given by

31 $$\begin{aligned} \frac{1}{n}\sum _{i} f_{z_0}(\sigma _i)\approx \frac{1}{\pi }\int _\mathbb {D}f_{z_0}(z){\text {d}}\!{}z, \end{aligned}$$

where $\mathbb {D}$ denotes the unit disk of the complex plane.

### Proposition 4

Let $k\in \mathbb {N}$ and $z_1, \dots , z_k\in \mathbb {C}$ be such that $\left|z_j\right|^2=1$ for all $j\in [k]$, and let $f^{(1)},\dots ,f^{(k)}$ be smooth compactly supported test functions. Denote the eigenvalues of an i.i.d. matrix $X$ satisfying Assumptions (A)–(B) and a corresponding real or complex Ginibre matrix $\widetilde{X}$ by $\{\sigma _i\}_{i=1}^n$, $\{\widetilde{\sigma }_i\}_{i=1}^n$. Then we have the bound

32 $$\begin{aligned}&{{\,\mathrm{\mathbf {E}}\,}}\Biggl [ \prod _{j=1}^k\left( \frac{1}{n}\sum _{i=1}^n f^{(j)}_{z_j}(\sigma _i) -\frac{1}{\pi } \int _\mathbb {D}f^{(j)}_{z_j}(z) {\text {d}}\!{}z \right) \nonumber \\&\qquad \qquad - \prod _{j=1}^k\left( \frac{1}{n}\sum _{i=1}^n f^{(j)}_{z_j} (\widetilde{\sigma }_i)- \frac{1}{\pi }\int _\mathbb {D}f^{(j)}_{z_j}(z) {\text {d}}\!{}z \right) \Biggr ] = \mathcal {O}( n^{-c(k)}), \end{aligned}$$

for some small constant $c(k)>0$, where the implicit multiplicative constant in $\mathcal {O}(\cdot )$ depends on the norms $\Vert \varDelta f^{(j)}\Vert _1$, $j=1,2, \ldots , k$.

### Proof of Theorem 1

Theorem 1 follows directly from Proposition 4 by the definition of the $k$-point correlation function in (6), the exclusion-inclusion principle and the bound

$$\begin{aligned} \left| { \frac{1}{\pi }\int _\mathbb {D}f_{z_0}(z) {\text {d}}\!{}z}\right| \lesssim 1. \end{aligned}$$

$$\square $$

The remainder of this section is devoted to the proof of Proposition 4. We now fix some $k\in \mathbb {N}$ and some $z_1,\dots ,z_k,f^{(1)},\dots ,f^{(k)}$ as in Proposition 4. All subsequent estimates in this section, also if not explicitly stated, hold true uniformly for any $z$ in an order $n^{-1/2}$-neighborhood of $z_1,\dots , z_k$. In order to prove (32), we use Girko’s formula (14) to write

33 $$\begin{aligned} \frac{1}{n}\sum _{i=1}^n f^{(j)}_{z_j}(\sigma _i)- \frac{1}{\pi }\int _\mathbb {D}f^{(j)}_{z_j}(z) {\text {d}}\!{}z =I_1^{(j)}+I_2^{(j)}+I_3^{(j)}+I_4^{(j)}, \end{aligned}$$

where

$$\begin{aligned} I_1^{(j)}&:= \frac{1}{4\pi n}\int _\mathbb {C}\varDelta f^{(j)}_{z_j}(z)\log \left|\det (H^z-\text {i}T)\right| {\text {d}}\!{}z \\ I_2^{(j)}&:=-\frac{1}{2\pi }\int _\mathbb {C}\varDelta f^{(j)}_{z_j}(z)\int _0^{\eta _0} \left[ \langle \mathfrak {I}G^z(\text {i}\eta )\rangle -\mathfrak {I}\widehat{m}^z(\text {i}\eta )\right] \, {\text {d}}\!{}\eta {\text {d}}\!{}z \\ I_3^{(j)}&:= -\frac{1}{2\pi }\int _\mathbb {C}\varDelta f^{(j)}_{z_j}(z)\int _{\eta _0}^{T} \left[ \langle \mathfrak {I}G^z(\text {i}\eta )\rangle -\mathfrak {I}\widehat{m}^z(\text {i}\eta )\right] \, {\text {d}}\!{}\eta {\text {d}}\!{}z \\ I_4^{(j)}&:= +\frac{1}{2\pi }\int _\mathbb {C}\varDelta f^{(j)}_{z_j}(z)\int _T^{+\infty } \left( \mathfrak {I}\widehat{m}^z(i\eta )-\frac{1}{\eta +1}\right) {\text {d}}\!{}\eta {\text {d}}\!{}z , \end{aligned}$$

with $\eta _0:= n^{-3/4-\delta }$, for some small fixed $\delta >0$, and for some very large $T>0$, say $T:= n^{100}$. We define $\widetilde{I}_1^{(j)}$, $\widetilde{I}_2^{(j)}$, $\widetilde{I}_3^{(j)}$, $\widetilde{I}_4^{(j)}$ analogously for the Ginibre ensemble by replacing $H^z$ by $\widetilde{H}^z$ and $G^z$ by $\widetilde{G}^z$.

### Proof of Proposition 4

The first step in the proof of Proposition 4 is the reduction to a corresponding statement about the $I_3$-part in (33), as summarized in the following lemma.

### Lemma 1

Let $k\ge 1$, let $I_3^{(1)},\dots , I_3^{(k)}$ be the integrals defined in (33), with $\eta _0=n^{-3/4-\delta }$, for some small fixed $\delta >0$, and let $\widetilde{I}_3^{(1)}, \dots , \widetilde{I}_3^{(k)}$ be defined as in (33) replacing $m^z$ with $\widetilde{m}^z$. Then,

34 $$\begin{aligned}&{{\,\mathrm{\mathbf {E}}\,}}\Biggl [ \prod _{j=1}^k\left( \frac{1}{n}\sum _{i=1}^n f_{z_j}^{(j)}(\sigma _i) - \frac{1}{\pi }\int _\mathbb {D}f_{z_j}^{(j)}(z) {\text {d}}\!{}z \right) \nonumber \\&\qquad \qquad \qquad - \prod _{j=1}^k\left( \frac{1}{n}\sum _{i=1}^n f_{z_j}^{(j)} (\widetilde{\sigma }_i)- \frac{1}{\pi }\int _\mathbb {D}f_{z_j}^{(j)}(z) {\text {d}}\!{}z \right) \Biggr ] \nonumber \\&\qquad = {{\,\mathrm{\mathbf {E}}\,}}\left[ \prod _{j=1}^k I_3^{(j)}-\prod _{j=1}^k \widetilde{I}_3^{(j)}\right] + \mathcal {O}\left( n^{-c_2(k,\delta )}\right) , \end{aligned}$$

for some small constant $c_2(k,\delta )>0$.

In order to conclude the proof of Proposition 4, due to Lemma 1, it only remains to prove that

35 $$\begin{aligned} {{\,\mathrm{\mathbf {E}}\,}}\left[ \prod _{j=1}^k I_3^{(j)}-\prod _{j=1}^k \widetilde{I}_3^{(j)}\right] =\mathcal {O}\left( n^{-c(k)}\right) , \end{aligned}$$

for any fixed $k$ with some small constant $c(k)>0$, where we recall the definition of $I_3$ and the corresponding $\widetilde{I}_3$ for Ginibre from (33). The proof of (35) is similar to the Green function comparison proof in Proposition 3 but more involved due to the fact that we compare products of resolvents and that we have an additional $\eta$-integration. Here we define the observable

36 $$\begin{aligned} Z_t := \prod _{j\in [k]} I_3^{(j)}(t):=\prod _{j\in [k]} \biggl (-\frac{1}{2\pi }\int _\mathbb {C}\varDelta f^{(j)}_{z_j}(z)\int _{\eta _0}^T \mathfrak {I}\langle G^z_t(\text {i}\eta )-M^z(\text {i}\eta )\rangle {\text {d}}\!{}\eta {\text {d}}\!{}z\biggr ), \end{aligned}$$

where we recall that $G_t^z(w):= (H^z_t-w)^{-1}$ with $H^z_t=H_t$ as in (20). $\square$

### Lemma 2

For any $n^{-1}\le \eta _0\le n^{-3/4}$ and $T=n^{100}$ and any small $\xi >0$ it holds that

37 $$\begin{aligned} \left|{{\,\mathrm{\mathbf {E}}\,}}[Z_{t_2}- Z_{t_1}]\right|\lesssim \Bigl (e^{-3t_0/2}-e^{-3t_1/2}\Bigr ) \frac{n^\xi }{n^{5/2}\eta _0^3}\prod _j \Vert \varDelta f^{(j)}\Vert _1 \end{aligned}$$

uniformly in $0\le t_1<t_2\le +\infty$ with the convention that $e^{-\infty }=0$.

Since $Z_0=\prod _j I_3^{(j)}$ and $Z_\infty =\prod _j \widetilde{I}_3^{(j)}$, the proof of Proposition 4 follows directly from (35), modulo the proofs of Lemmata 1–2 that will be given in the next two subsections. $\square$

### Proof of Lemma 1

In order to estimate the probability that there exists an eigenvalue of $H^z$ very close to zero, we use the following proposition that has been proven in [3, Prop. 5.7] adapting the proof of [9, Lemma 4.12].

#### Proposition 5

Under Assumption (B) there exists a constant $C>0$, depending only on $\alpha$, such that

38 $$\begin{aligned} {{\,\mathrm{\mathbf {P}}\,}}\left( \min _{i\in [2n]}\left|\lambda _i^z\right|\le \frac{u}{n} \right) \le C u^\frac{2\alpha }{1+\alpha }n^{\beta +1}, \end{aligned}$$

for all $u>0$ and $z\in \mathbb {C}$.

In the following lemma we prove a very high probability bound for $I_1^{(j)}$, $I_2^{(j)}$, $I_3^{(j)}$, $I_4^{(j)}$. The same bounds hold true for $\widetilde{I}_1^{(j)}$, $\widetilde{I}_2^{(j)}$, $\widetilde{I}_3^{(j)}$, $\widetilde{I}_4^{(j)}$ as well. These bounds in the bulk regime were already proven in [3, Proof of Theorem 2.5] the current edge regime is analogous, so we only provide a sketch of the proof for completeness.

#### Lemma 3

For any $j\in [k]$ the bounds

39 $$\begin{aligned} \left| {I_1^{(j)}}\right| \le \frac{n^{1+\xi }\Vert \varDelta f^{(j)}\Vert _1}{T^2}, \quad \left| {I_2^{(j)}}\right| + \left| {I_3^{(j)}}\right| \le n^\xi \Vert \varDelta f^{(j)}\Vert _1 , \quad \left| {I_4^{(j)}}\right| \le \frac{n\Vert \varDelta f^{(j)}\Vert _1}{T}, \end{aligned}$$

hold with very high probability for any $\xi >0$. The bounds analogous to (39) also hold for $\widetilde{I}_l^{(j)}$.

#### Proof

For notational convenience we do not carry the $j$-dependence of $I_l^{(j)}$ and $f^{(j)}$, and the dependence of $\lambda _i,H,G,M,\widehat{m}$ on $z$ within this proof. Using that

$$\begin{aligned} \log \left|\det (H -\text {i}T)\right|= 2n\log T+\sum _{j\in [n]}\log \left( 1+\frac{\lambda _j^{2}}{T^2}\right) , \end{aligned}$$

we easily estimate $\left|I_1\right|$ as follows

$$\begin{aligned} \left|I_1\right|&=\left| \frac{1}{4\pi n}\int _\mathbb {C}\varDelta f_{z_j}(z)\log \left|\det (H-\text {i}T)\right| {\text {d}}\!{}z \right|\\&\lesssim \frac{1}{n}\int _\mathbb {C}\left|\varDelta f_{z_j}(z)\right|\frac{{{\,\mathrm{Tr}\,}}H^2}{T^2} {\text {d}}\!{}z\lesssim \frac{n^{1+\xi }\Vert \varDelta f\Vert _1}{T^2}, \end{aligned}$$

for any $\xi >0$ with very high probability owing to the high moment bound (3). By (9) it follows that $\left|\mathfrak {I}\widehat{m}^z(\text {i}\eta )-(\eta +1)^{-1}\right|\sim \eta ^{-2}$ for large $\eta$, proving also the bound on $I_4$ in (39). The bound for $I_3$ follows immediately from the averaged local law in (13).

For the $I_2$ estimate we split the $\eta$-integral of $\mathfrak {I}m^z(\text {i}\eta )-\mathfrak {I}\widehat{m}^z(\text {i}\eta )$ in $I_2$ as follows

40 $$\begin{aligned}&\int _0^{\eta _0} \mathfrak {I}\langle G^z(\text {i}\eta )-M^z(\text {i}\eta )\rangle {\text {d}}\!{}\eta \nonumber \\&\qquad =\frac{1}{n}\sum _{\left|\lambda _i\right|< n^{-l}}\log \left( 1+\frac{\eta _0^2}{\lambda _i^2}\right) +\frac{1}{n} \sum _{\left|\lambda _i\right|\ge n^{-l}}\log \left( 1+\frac{\eta _0^2}{\lambda _i^2}\right) -\int _0^{\eta _0} \mathfrak {I}\widehat{m}^z(\text {i}\eta ) {\text {d}}\!{}\eta , \end{aligned}$$

where $l\in \mathbb {N}$ is a large fixed integer. Using (10) we find that the third term in (40) is bounded by $n^{-1-\delta }$. Choosing $l$ large enough, it follows, as in [3, Eq. (5.35)] using the bound (38) that

41 $$\begin{aligned} \frac{1}{n}\sum _{\left|\lambda _i\right|< n^{-l}}\log \left( 1+\frac{\eta _0^2}{\lambda _i^2}\right) \le n^{-1+\xi }, \end{aligned}$$

with very high probability for any $\xi >0$. Alternatively, this bound also follows from (5) without Assumption (B), circumventing Proposition 5, see Remark 1. For the second term in (40) we define $\eta _1:= n^{-3/4+\xi }$ with some very small $\xi >0$ and using $\log (1+x)\le x$ we write

42 $$\begin{aligned} \sum _{\left|\lambda _i\right|\ge n^{-l}}\log \left( 1+\frac{\eta _0^2}{\lambda _i^2}\right)&= \sum _{ n^{-l}\le \left|\lambda _i\right|\le n^{\delta /2} \eta _0}\log \left( 1+\frac{\eta _0^2}{\lambda _i^2}\right) + \eta _0^2 \sum _{\left|\lambda _i\right|\ge n^{\delta /2}\eta _0} \frac{1}{\lambda _i^2} \nonumber \\&\lesssim \left|\{i\vert \left|\lambda _i\right|< n^{\delta /2}\eta _0\}\right| \cdot \log n + \eta _0^2 \sum _{\left|\lambda _i\right|\ge n^{\delta /2}\eta _0} \frac{1}{\lambda _i^2} \nonumber \\&\lesssim (\log n) n^{4\xi /3} + \frac{\eta _0^2 n^{\delta + 2\xi }}{\eta _1} \sum _{\left|\lambda _i\right|\ge n^{\delta /2}\eta _0} \frac{\eta _1}{\lambda _i^2+\eta _1^2} \nonumber \\&\lesssim (\log n) n^{4\xi /3}+ n^{1-\delta }\eta _1 \langle \mathfrak {I}G^z(\text {i}\eta _1)\rangle \le n^{2\xi } + n^{-\delta +2\xi } \end{aligned}$$

by the averaged local law in (13), and $\langle \mathfrak {I}M^z(\text {i}\eta _1)\rangle \lesssim \eta _1^{1/3}$ from (10). Here from the second to third line in (42) we used that

43 $$\begin{aligned} \left|\{i\vert \left|\lambda _i\right|\le n^{\delta /2}\eta _0\}\right|\le \sum _i \frac{\eta _1^2}{\lambda _i^2 + \eta _1^2} =n\eta _1 \langle \mathfrak {I}G^z(\text {i}\eta _1)\rangle \le n^{4\xi /3}, \end{aligned}$$

again by the local law. By redefining $\xi$, this concludes the high probability bound on $I_2$ in (39), and thereby the proof of the lemma. $\square$

In the following lemma we prove an improved bound for $I_2^{(j)}$, compared with (39), which holds true only in expectation. The main input of the following lemma is the stronger lower tail estimate on $\lambda _i$, in the regime $\left|\lambda _i\right|\ge n^{-l}$, from (15) instead of (43).

#### Lemma 4

Let $I_2^{(j)}$ be defined in (33), then

44 $$\begin{aligned} {{\,\mathrm{\mathbf {E}}\,}}\left|I_2^{(j)}\right|\lesssim n^{-\delta /3} \Vert \varDelta f^{(j)} \Vert _1, \end{aligned}$$

for any $$j\in \{1,\dots , k\}$$.

#### Proof

We split the $\eta$-integral of $\mathfrak {I}m^z(\text {i}\eta )-\mathfrak {I}\widehat{m}^z(\text {i}\eta )$ as in (40). The third term in the r.h.s. of (40) is of order $n^{-1-4\delta /3}$. Then, we estimate the first term in the r.h.s. of (40) in terms of the smallest (in absolute value) eigenvalue $\lambda _{n+1}$ as

45 $$\begin{aligned} {{\,\mathrm{\mathbf {E}}\,}}\left[ \frac{1}{n}\sum _{\left|\lambda _i\right|< n^{-l}} \log \left( 1+\frac{\eta _0^2}{\lambda _i^2}\right) \right]&\le {{\,\mathrm{\mathbf {E}}\,}}\left[ \log \left( 1+\frac{\eta _0^2}{\lambda _{n+1}^2}\right) \mathbf {1}(\lambda _{n+1}\le n^{-l})\right] \nonumber \\&\lesssim {{\,\mathrm{\mathbf {E}}\,}}[\left|\log \lambda _{n+1}\right| \mathbf {1}(\lambda _{n+1}\le n^{-l})] \nonumber \\&=\int _{l\log n}^{+\infty }{{\,\mathrm{\mathbf {P}}\,}}(\lambda _{n+1}\le e^{-t}) \, {\text {d}}\!{}t\lesssim n^{\beta +1+\frac{2\alpha }{1+\alpha }} n^{-\frac{2\alpha l}{1+\alpha }}, \end{aligned}$$

where in the last inequality we use (38) with $u=e^{-t} n$. Note that by (15) it follows that

46 $$\begin{aligned} {{\,\mathrm{\mathbf {E}}\,}}\left| \{i:\left|\lambda _i\right|\le n^{\delta /2}\eta _0\} \right|\lesssim n^{-\delta /2}. \end{aligned}$$

Hence, by (46), using similar computations to (42), we conclude that

47 $$\begin{aligned} {{\,\mathrm{\mathbf {E}}\,}}\left[ \frac{1}{n} \sum _{\left|\lambda _i\right|\ge n^{-l}}\log \left( 1+\frac{\eta _0^2}{\lambda _i^2}\right) \right] \lesssim \frac{\log n}{n^{1+\delta /2}}. \end{aligned}$$

Note that the only difference to prove (47) respect to (42) is that the first term in the first line of the r.h.s. of (42) is estimated using (46) instead of (43). Finally, choosing $l\ge \alpha ^{-1} (3+\beta )(1+\alpha )+2$, and combining (45), (47) we conclude (44). $\square$

Equipped with Lemmata 3–4, we now present the proof of Lemma 1.

#### Proof of Lemma 1

Using the definitions for $I_1^{(j)}, I_2^{(j)},I_3^{(j)}, I_4^{(j)}$ in (33), and similar definitions for $\widetilde{I}_1^{(j)}, \widetilde{I}_2^{(j)},\widetilde{I}_3^{(j)}, \widetilde{I}_4^{(j)}$, we conclude that

$$\begin{aligned}&{{\,\mathrm{\mathbf {E}}\,}}\Biggl [ \prod _{j=1}^k\left( \frac{1}{n}\sum _{i=1}^n f_{z_j}^{(j)}(\sigma _i) - \frac{1}{\pi }\int _\mathbb {D}f_{z_j}^{(j)}(z) {\text {d}}\!{}z \right) \\&\qquad \qquad \qquad -\prod _{j=1}^k\left( \frac{1}{n}\sum _{i=1}^n f_{z_j}^{(j)} (\widetilde{\sigma }_i)- \frac{1}{\pi }\int _\mathbb {D}f_{z_j}^{(j)}(z) {\text {d}}\!{}z \right) \Biggr ] \\&\qquad = {{\,\mathrm{\mathbf {E}}\,}}\left[ \prod _{j=1}^k\left( I_1^{(j)}+ I_2^{(j)}+I_3^{(j)}+ I_4^{(j)}\right) - \prod _{j=1}^k\left( \widetilde{I}_1^{(j)}+ \widetilde{I}_2^{(j)}+\widetilde{I}_3^{(j)} + \widetilde{I}_4^{(j)}\right) \right] \\&\qquad = {{\,\mathrm{\mathbf {E}}\,}}\left[ \prod _{j=1}^k I_3^{(j)}-\prod _{j=1}^k \widetilde{I}_3^{(j)}\right] +\sum _{\begin{array}{c} j_1+j_2+j_3+j_4=k\\ j_i\ge 0, \, j_3<k \end{array}} {{\,\mathrm{\mathbf {E}}\,}}\prod _{\begin{array}{c} i_l=1,\\ l=1,2,3,4 \end{array}}^{j_l} I_1^{(i_1)} I_2^{(i_2)} I_3^{(i_3)} I_4^{(i_4)} \\&\qquad \quad -\sum _{\begin{array}{c} j_1+j_2+j_3+j_4=k,\\ j_i\ge 0, \, j_3<k \end{array}} {{\,\mathrm{\mathbf {E}}\,}}\prod _{\begin{array}{c} i_l=1\\ l=1,2,3,4 \end{array}}^{j_l} \widetilde{I}_1^{(i_1)} \widetilde{I}_2^{(i_2)} \widetilde{I}_3^{(i_3)} \widetilde{I}_4^{(i_4)}. \end{aligned}$$

Then, if $j_2\ge 1$, by Lemmas 3 and 4, using that $T=n^{100}$ in the definition of $I_1^{(j)},\dots , I_4^{(j)}$ in (33), it follows that

$$\begin{aligned} {{\,\mathrm{\mathbf {E}}\,}}\prod _{\begin{array}{c} i_l=1,\\ l=1,2,3,4 \end{array}}^{j_l} I_1^{(i_1)} I_2^{(i_2)} I_3^{(i_3)} I_4^{(i_4)}\lesssim \frac{n^{j_1+j_4} n^{(k-j_4-1)\xi } \ \prod _{j=1}^k \Vert \varDelta f^{(j)}\Vert _1}{n^{\delta /3} T^{2j_1+j_4}}\le n^{-c_2(k,\delta )}, \end{aligned}$$

for any $j_1,j_3, j_4 \ge 0$, and a small constant $c(_2k,\delta )>0$ which only depends on $k, \delta$. If, instead, $j_2=0$, then at least one among $j_1$ and $j_4$ is not zero, since $0\le j_3\le k-1$ and $j_1+j_2+j_3+j_4=k$. Assume $j_1\ge 1$, the case $j_4\ge 1$ is completely analogous, then

$$\begin{aligned} {{\,\mathrm{\mathbf {E}}\,}}\prod _{\begin{array}{c} i_l=1,\\ l=1,2,3,4 \end{array}}^{j_l} I_1^{(i_1)} I_2^{(i_2)} I_3^{(i_3)} I_4^{(i_4)}\lesssim \frac{n^{j_1+j_4} n^{(k-j_4)\xi } \prod _{j=1}^k \Vert \varDelta f^{(j)}\Vert _1}{T^{2j_1+j_4}} \le n^{-c_2(k,\delta )}. \end{aligned}$$

Since similar bounds hold true for $\widetilde{I}_1^{(i_1)}, \widetilde{I}_2^{(i_2)}, \widetilde{I}_3^{(i_3)}, \widetilde{I}_4^{(i_4)}$ as well, the above inequalities conclude the proof of (34). $\square$

### Proof of Lemma 2

We begin with a lemma generalizing the bound in (39) to derivatives of $I_3^{(j)}$.

#### Lemma 5

Assume $n^{-1}\le \eta _0\le n^{-3/4}$ and fix $l\ge 0$, $j\in [k]$ and a double index $\alpha =(a,b)$ such that $a\ne b$. Then, for any choice of $\gamma _i\in \{\alpha ,\alpha '\}$ and any $\xi >0$ we have the bounds

48 $$\begin{aligned} \left| \partial _\gamma ^l I_3^{(j)}(t) \right|\lesssim \Vert \varDelta f^{(j)}\Vert _1 n^\xi \biggl ( \frac{1}{(n\eta _0)^{\min \{l,2\}}} + \mathbf {1}\bigl (a\equiv b+n\pmod {2n}\bigr ) \biggr ), \end{aligned}$$

where $\partial _\gamma ^l:=\partial _{\gamma _1}\dots \partial _{\gamma _l}$, with very high probability uniformly in $t\ge 0$.

#### Proof

We omit the $t$- and $z$-dependence of $G_t^z$, $\widehat{m}^z$ within this proof since all bounds hold uniformly in $t\ge 0$ and $\left|z-z_j\right|\lesssim n^{-1/2}$. We also omit the $\eta$-argument from these functions, but the $\eta$-dependence of all estimates will explicitly be indicated. Note that the $l=0$ case was already proven in (39). We now separately consider the remaining cases $l=1$ and $l\ge 2$. For notational simplicity we neglect the $n^\xi$ multiplicative error factors (with arbitrarily small exponents $\xi >0$) applications of the local law  (13) within the proof. In particular we will repeatedly use (13) in the form

49 $$\begin{aligned} \left|G_{ba}\right|&\lesssim {\left\{ \begin{array}{ll} 1, &{} a\equiv b+n\pmod {2n},\\ \psi , &{} a\not \equiv b+n \pmod {2n},\end{array}\right. } \quad G_{bb}=\widehat{m} + \mathcal {O}(\psi ),\nonumber \\ \left|\widehat{m}\right|&\lesssim \min \{1,\eta ^{1/3}+n^{-1/4}\}, \end{aligned}$$

where we defined the parameter

$$\begin{aligned} \psi := \frac{1}{n\eta }+\frac{1}{n^{1/2}\eta ^{1/3}}. \end{aligned}$$

#### Case $l=1$

This follows directly from

$$\begin{aligned} \left|\int _{\eta _0}^T \langle G \varDelta ^{ab} G\rangle {\text {d}}\!{}\eta \right|&= \left|\frac{1}{n} \int _{\eta _0}^T G^2_{ba}{\text {d}}\!{}\eta \right| = \frac{\left|G(\text {i}T)_{ab}-G(\text {i}\eta _0)_{ab}\right|}{n}\\&\lesssim \frac{1}{n^2\eta _0} + \frac{1}{n}\mathbf {1}\bigl (a\equiv b+n\pmod {2n}\bigr ), \end{aligned}$$

where in the last step we used $\Vert G(\text {i}T)\Vert \le T^{-1}=n^{-100}$ and (49). Since this bound is uniform in $z$ we may bound the remaining integral by $n\Vert \varDelta f^{(j)}\Vert _1$, proving (48).

#### Case $l\ge 2$

For the case $l\ge 2$ there are many assignments of $\gamma _i$’s to consider, e.g.

$$\begin{aligned}&\langle G\varDelta ^{ab}G\varDelta ^{ab}G\rangle = \frac{1}{n} \sum _c G_{ca} G_{ba} G_{bc}, \quad \langle G\varDelta ^{ab}G\varDelta ^{ba}G\rangle = \frac{1}{n} \sum _c G_{ca} G_{bb} G_{ac}, \\&\langle G\varDelta ^{ab}G\varDelta ^{ba}G\varDelta ^{ab}G\rangle = \frac{1}{n} \sum _c G_{ca} G_{bb} G_{aa} G_{bc},\\&\langle G\varDelta ^{ab}G\varDelta ^{ba}G\varDelta ^{ba}G\rangle = \frac{1}{n} \sum _c G_{ca} G_{bb} G_{ab} G_{ac} \end{aligned}$$

but all are of the form that there are two $G$-factors carrying the independent summation index $c$. In the case that $a\equiv b+n\pmod {2n}$ we simply bound all remaining $G$-factors by $1$ using (49) and use a simple Cauchy-Schwarz inequality to obtain

50 $$\begin{aligned} \left|\partial _\gamma ^l I_3^{(j)}\right| \lesssim \int _\mathbb {C}\left|\varDelta f_{z_j}^{(j)}(z)\right| \frac{1}{n}\int _{\eta _0}^T \sum _c \Bigl (\left|G_{cb}\right|^2+\left|G_{ca}\right|^2 \Bigr ){\text {d}}\!{}\eta {\text {d}}\!{}z. \end{aligned}$$

Now it follows from the Ward-identity

51 $$\begin{aligned} GG^*= G^*G = \frac{\mathfrak {I}G}{\eta } \end{aligned}$$

and the very crude bound $\left|G_{aa}\right|\lesssim 1$ from  (49) and $\left|\widehat{m}\right|\lesssim 1$, that

$$\begin{aligned} \int _{\eta _0}^T \sum _c \Bigl (\left|G_{cb}\right|^2+\left|G_{ca}\right|^2 \Bigr ){\text {d}}\!{}\eta = \int _{\eta _0}^T \frac{\left|(\mathfrak {I}G)_{aa}\right|+\left|(\mathfrak {I}G)_{bb}\right|}{\eta }{\text {d}}\!{}\eta \lesssim \int _{\eta _0}^T \frac{1}{\eta }{\text {d}}\!{}\eta \lesssim \log n. \end{aligned}$$

By estimating the remaining $z$-integral in (50) by $n\Vert \varDelta f^{(j)}\Vert$ the claimed bound in  (48) for $a=b+n\pmod {2n}$ follows.

In the case $a\not \equiv b+n\pmod {2n}$ we can use  (49) to gain a factor of $\psi$ for some $G_{ab}$ or $G_{bb}-\widehat{m}$ in all assignments except for the one in which all but two $G$-factors are diagonal, and those $G_{aa},G_{bb}$-factors are replaced by $\widehat{m}$. For example, we would expand

$$\begin{aligned} G_{ca}G_{bb}G_{aa}G_{bc}=\widehat{m}^2 G_{ca}G_{bc} + \widehat{m} G_{ca}G_{bc} \mathcal {O}(\psi ) + G_{ca}G_{bc} \mathcal {O}(\psi ^2), \end{aligned}$$

where in all but the first term we gained at least a factor of $\psi$. Using Cauchy-Schwarz as before we thus have the bound

52 $$\begin{aligned} \left|\partial _\gamma ^l I_3^{(j)}\right|&\lesssim \int _\mathbb {C}\frac{\left|\varDelta f_{z_j}^{(j)}(z)\right|}{n} \biggl (\int _{\eta _0}^T \psi \sum _c \Bigl (\left|G_{cb}\right|^2+\left|G_{ca}\right|^2 \Bigr ){\text {d}}\!{}\eta \nonumber \\&\qquad +\left|\int _{\eta _0}^T (\widehat{m})^{l-1} (G^2)_{aa}{\text {d}}\!{}\eta \right|+\left|\int _{\eta _0}^T (\widehat{m})^{l-1} (G^2)_{ab}{\text {d}}\!{}\eta \right| \biggr ){\text {d}}\!{}z, \end{aligned}$$

where strictly speaking, the second and third terms are only present for even, or respectively odd, $l$. For the first term in (52) we again proceed by applying the Ward identity (51), and (49) to obtain the bound

$$\begin{aligned} \int _{\eta _0}^T \psi \sum _c \Bigl (\left|G_{cb}\right|^2+\left|G_{ca}\right|^2 \Bigr ){\text {d}}\!{}\eta&= \int _{\eta _0}^T \psi \frac{\left|(\mathfrak {I}G)_{aa}\right|+\left|(\mathfrak {I}G)_{bb}\right|}{\eta }{\text {d}}\!{}\eta \\&\lesssim \int _{\eta _0}^T \frac{\psi (\psi +\eta ^{1/3})}{\eta }{\text {d}}\!{}\eta \lesssim \frac{\log n}{(n\eta _0)^2}. \end{aligned}$$

For the second and third terms in (52) we use $\text {i}G^2=G'$, where prime denotes $\partial _\eta$, and integration by parts, $\left|\widehat{m}'\right|\lesssim \eta ^{-2/3}$ from  (12), and (49) to obtain the bounds

$$\begin{aligned} \left|\int _{\eta _0}^T (\widehat{m})^{l-1} (G^2)_{aa}{\text {d}}\!{}\eta \right|&\lesssim \left|\int _{\eta _0}^T \widehat{m}'(\widehat{m})^{l-2} G_{aa}{\text {d}}\!{}\eta \right| \\&\qquad +\left|(\widehat{m}(\text {i}\eta _0))^{l-1} G(\text {i}\eta _0)_{aa}\right| + \left|(\widehat{m}(\text {i}T))^{l-1} G(\text {i}T)_{aa}\right| \\&\lesssim \left|\int _{\eta _0}^T \widehat{m}' (\widehat{m})^{l-1}{\text {d}}\!{}\eta \right| +\int _{\eta _0}^T \left|\widehat{m}'\right|\psi {\text {d}}\!{}\eta + \frac{1}{n^{1/4} (n\eta _0) }\\&\lesssim \frac{\log n}{n^{1/4} (n\eta _0) } \end{aligned}$$

and

$$\begin{aligned} \left|\int _{\eta _0}^T (\widehat{m})^{l-1} (G^2)_{ab}{\text {d}}\!{}\eta \right|&\lesssim \left|\int _{\eta _0}^T \widehat{m}' (\widehat{m})^{l-2} G_{ab}{\text {d}}\!{}\eta \right| \\&\qquad + \left|(\widehat{m}(\text {i}\eta _0))^{l-1} G(\text {i}\eta _0)_{ab}\right| + \left|(\widehat{m}(\text {i}T))^{l-1} G(\text {i}T)_{ab}\right| \\&\lesssim \int _{\eta _0}^T \left|\widehat{m}'\right|\psi {\text {d}}\!{}\eta + \frac{1}{n^{1/4} (n\eta _0) } \lesssim \frac{\log n}{n^{1/4} (n\eta _0) }. \end{aligned}$$

In the explicit deterministic term we performed an integration and estimated

$$\begin{aligned} \left|\int _{\eta _0}^T \widehat{m}' (\widehat{m})^{l-1}{\text {d}}\!{}\eta \right| \lesssim \left|\widehat{m}(\text {i}\eta _0)\right|^l+\left|\widehat{m}(\text {i}T)\right|^l \lesssim n^{-l/4}+n^{-100}\le n^{-1/2}. \end{aligned}$$

The claim (48) for $l\ge 2$ and $a\not \equiv b+n\pmod {2n}$ now follows from estimating the remaining $z$-integral in (52) by $n\Vert \varDelta f^{(j)}\Vert _1$. $\square$

##### Proof of Lemma 2

By (20) and Ito’s Lemma it follows that

53 $$\begin{aligned} {{\,\mathrm{\mathbf {E}}\,}}\frac{{\text {d}}\!{}Z_t}{{\text {d}}\!{}t}={{\,\mathrm{\mathbf {E}}\,}}\left[ -\frac{1}{2}\sum _\alpha h_\alpha (t) \partial _\alpha Z_t+\frac{1}{2}\sum _{\alpha ,\beta }\kappa _t(\alpha ,\beta ) \partial _\alpha \partial _\beta Z_t\right] , \end{aligned}$$

where we recall the definition of $\kappa _t$ in 23. In fact, the point-wise estimate from Lemma 5 gives a sufficiently strong bound for most terms in the cumulant expansion, the few remaining terms will be computed more carefully.

In the cumulant expansion (25) of (53) the second order terms cancel exactly and we now separately estimate the third-, fourth- and higher order terms.

#### Order three terms

For the third order, when computing $\partial _\alpha \partial _{\beta _1}\partial _{\beta _2} Z_t$ through the Leibniz rule we have to consider all possible assignments of derivatives $\partial _\alpha ,\partial _{\beta _1},\partial _{\beta _2}$ to the factors $I_3^{(1)},\dots ,I_3^{(k)}$. Since the particular functions $f^{(j)}$ and complex parameters $z_j$ play no role in the argument, there is no loss in generality in considering only the assignments

54 $$\begin{aligned}&\Bigl (\partial _{\alpha ,\beta _1,\beta _2} I_3^{(1)}\Bigr )\prod _{j>1} I_3^{(j)},\;\; \Bigl (\partial _{\alpha ,\beta _1} I_3^{(1)}\Bigr )\Bigl (\partial _{\beta _2} I_3^{(2)}\Bigr ) \prod _{j>2} I_3^{(j)},\nonumber \\&\Bigl (\partial _\alpha I_3^{(1)}\Bigr )\Bigl (\partial _{\beta _1} I_3^{(2)}\Bigr ) \Bigl (\partial _{\beta _2} I_3^{(3)}\Bigr ) \prod _{j>3} I_3^{(j)} \end{aligned}$$

for the second and third term of which we obtain a bound of

$$\begin{aligned}&n^{\xi -3/2}e^{-3t/2} \biggl (\sum _{a\equiv b+n} \prod _j \Vert \varDelta f^{(j)}\Vert _1 + \sum _{a\not \equiv b+n} \prod _j \Vert \varDelta f^{(j)}\Vert _1 \frac{1}{(n\eta _0)^3} \biggr )\\&\qquad \lesssim \frac{n^\xi e^{-3t/2}}{n^{5/2}\eta _0^3}\prod _j \Vert \varDelta f^{(j)}\Vert _1 \end{aligned}$$

using Lemma 5 and the cumulant scaling (24). Note that the condition $a\ne b$ in the lemma is ensured by the fact that for $a=b$ the cumulants $\kappa _t(\alpha ,\beta _1,\dots )$ vanish.

The first term in (54) requires an additional argument. We write out all possible index allocations and claim that ultimately we obtain the same bound, as for the other two terms in (54), i.e.

55 $$\begin{aligned} \left|\sum _{\alpha \beta _1\beta _2} \kappa _t(\alpha ,\beta _1,\beta _2) \partial _\alpha \partial _{\beta _1}\partial _{\beta _2} I_3^{(1)}\right|&\lesssim \frac{e^{-3t/2}}{n^{3/2}}\int _\mathbb {C}\frac{\left|\varDelta f_{z_1}^{(1)}\right|}{n} J_3 {\text {d}}\!{}z \nonumber \\&\lesssim \frac{n^\xi e^{-3t/2}}{n^{5/2}\eta _0^3} \Vert \varDelta f^{(1)}\Vert _1 \end{aligned}$$

where

56 $$\begin{aligned} J_3&:= \left|\int _{\eta _0}^T \sum _{ab} (G^2)_{ab} G_{ab} G_{ab}{\text {d}}\!{}\eta \right| +\left|\int _{\eta _0}^T \sum _{ab} (G^2)_{aa} G_{bb} G_{ab}{\text {d}}\!{}\eta \right|\nonumber \\&\qquad +\left|\int _{\eta _0}^T \sum _{ab} (G^2)_{ab} G_{aa} G_{bb}{\text {d}}\!{}\eta \right|. \end{aligned}$$

##### Proof of Eq. (55)

Compared to the previous bound in Lemma 5 we now exploit the $a,b$ summation via the isotropic structure of the bound in the local law (59). We have the simple bounds

57 $$\begin{aligned} \frac{\left|\langle \varvec{x},\mathfrak {I}G\varvec{x}\rangle \right|}{\Vert \varvec{x}\Vert ^2}&\lesssim \left|\widehat{m}\right|+n^\xi \psi \lesssim n\eta \psi ^2,\nonumber \\ \left|\langle \varvec{x}, G^2 \varvec{y}\rangle \right|&\le \frac{1}{\eta } \sqrt{\langle \varvec{x},\mathfrak {I}G\varvec{x}\rangle \langle \varvec{y},\mathfrak {I}G\varvec{y}\rangle }\lesssim n^\xi \Vert \varvec{x}\Vert \Vert \varvec{y}\Vert n\psi ^2 \end{aligned}$$

as a consequence of the Ward identity (51) and using (13) and (10). For the first term in (56) we can thus use (57) and  (51) to obtain

$$\begin{aligned} \left|\int _{\eta _0}^T \sum _{ab} (G^2)_{ab} G_{ab} G_{ab}{\text {d}}\!{}\eta \right|&\lesssim n^\xi \int _{\eta _0}^T n \psi ^2 \sum _{ab}\left|G_{ab}\right|^2 {\text {d}}\!{}\eta \\&\lesssim n^\xi \int _{\eta _0}^T n \psi ^2 \sum _a\frac{(\mathfrak {I}G)_{aa}}{\eta } {\text {d}}\!{}\eta \\&\lesssim n^\xi \int _{\eta _0}^T n^3 \psi ^4{\text {d}}\!{}\eta \lesssim \frac{n^\xi }{n\eta _0^3}. \end{aligned}$$

For the second term in (56) we split $G_{bb}=\widehat{m}+ \mathcal {O}(\psi )$ and bound it by

$$\begin{aligned}&\left|\int _{\eta _0}^T \sum _{ab} (G^2)_{aa} G_{bb} G_{ab}{\text {d}}\!{}\eta \right| \\&\quad \lesssim n^\xi \int _{\eta _0}^T \psi \sum _{ab} \left| (G^2)_{aa} G_{ab}\right| {\text {d}}\!{}\eta + \left|\int _{\eta _0}^T \widehat{m} \sum _{a} (G^2)_{aa} \langle e_a,G\varvec{1}_{s(a)}\rangle {\text {d}}\!{}\eta \right|\\&\quad \lesssim n^\xi \int _{\eta _0}^T n^{3/2} \psi ^2 \biggl (\psi \sum _b \sqrt{\frac{(\mathfrak {I}G)_{bb}}{\eta }}+ \sqrt{\frac{ \langle \varvec{1}_+,\mathfrak {I}G\varvec{1}_+\rangle +\langle \varvec{1}_-,\mathfrak {I}G\varvec{1}_-\rangle }{\eta }}\biggr ){\text {d}}\!{}\eta \\&\quad \lesssim n^\xi \int _{\eta _0}^T \Bigl (n^{3} \psi ^4+n^{5/2}\psi ^3 \Bigr ) {\text {d}}\!{}\eta \lesssim \frac{n^\xi }{n\eta _0^3} \end{aligned}$$

where $e_a$ denotes the $a$-th standard basis vector,

58 $$\begin{aligned} \varvec{1}_+ := (1,\ldots ,1,0,\ldots ,0), \quad \varvec{1}_- := (0,\ldots ,0,1,\ldots ,1) \end{aligned}$$

are vectors of $n$ ones and zeros, respectively, of norm $\Vert \varvec{1}_\pm \Vert =\sqrt{n}$ and $s(a):=-$ for $a\le n$, and $s(a):=+$ for $a>n$. Here in the second step we used a Cauchy-Schwarz inequality for the $a$-summation in both integrals after estimating the $G^2$-terms using (57). Finally, for the third term in (56) we split both $G_{aa}=\widehat{m}+\mathcal {O}(\psi )$ and $G_{bb}=\widehat{m}+\mathcal {O}(\psi )$ to estimate

$$\begin{aligned}&\left|\int _{\eta _0}^T \sum _{ab} (G^2)_{ab} G_{aa} G_{bb}{\text {d}}\!{}\eta \right| \\&\quad \lesssim n^\xi \int _{\eta _0}^T n^3 \psi ^4{\text {d}}\!{}\eta + \sum _{a} \int _{\eta _0}^T \left| \widehat{m} \langle e_a, G^2 \varvec{1}_{s(a)}\rangle \psi \right| {\text {d}}\!{}\eta +\int _{\eta _0}^T \left|\widehat{m}^2 \langle \varvec{1}_+, G^2\varvec{1}_-\rangle \right|{\text {d}}\!{}\eta \\&\quad \lesssim \frac{n^\xi }{n\eta _0^3} +n^\xi \int _{\eta _0}^T n^{5/2}\psi ^3 {\text {d}}\!{}\eta +n^\xi \int _{\eta _0}^T \frac{n^2 \psi ^2}{1+\eta ^2}{\text {d}}\!{}\eta \lesssim \frac{n^\xi }{n\eta _0^3}, \end{aligned}$$

using (57). In the last integral we used that $\left|\widehat{m}\right|\lesssim (1+\eta )^{-1}$ to ensure the integrability in the large $\eta$-regime. Inserting these estimates on  (56) into (55) and estimating the remaining integral by $n\Vert \varDelta f^{(1)}\Vert _1$ completes the proof of (55). $\square$

#### Order four terms

For the fourth-order Leibniz rule we have to consider the assignments

$$\begin{aligned}&\Bigl (\partial _{\alpha ,\beta _1,\beta _2,\beta _3} I_3^{(1)}\Bigr )\prod _{j>1} I_3^{(j)},\;\; \Bigl (\partial _{\alpha ,\beta _1,\beta _2} I_3^{(1)}\Bigr ) \Bigl (\partial _{\beta _3} I_3^{(2)}\Bigr )\prod _{j>2} I_3^{(j)},\\&\Bigl (\partial _{\alpha ,\beta _1} I_3^{(1)}\Bigr ) \Bigl (\partial _{\beta _2,\beta _3} I_3^{(2)}\Bigr ) \prod _{j>2} I_3^{(j)}, \;\; \Bigl (\partial _{\alpha ,\beta _1} I_3^{(1)}\Bigr )\Bigl (\partial _{\beta _2} I_3^{(2)}\Bigr ) \Bigl (\partial _{\beta _3} I_3^{(3)}\Bigr ) \prod _{j>3} I_3^{(j)},\\&\Bigl (\partial _{\alpha ,\beta _1} I_3^{(1)}\Bigr )\Bigl (\partial _{\beta _2} I_3^{(2)}\Bigr ) \Bigl (\partial _{\beta _2} I_3^{(3)}\Bigr )\Bigl (\partial _{\beta _3} I_3^{(4)}\Bigr ) \prod _{j>4} I_3^{(j)}, \end{aligned}$$

for all of which we obtain a bound of

$$\begin{aligned} \frac{n^{\xi } e^{-2t}}{n^2\eta _0^2} \prod _j \Vert \varDelta f^{(j)}\Vert _1, \end{aligned}$$

again using Lemma 5 and (24).

#### Higher order terms

For terms order at least $5$, there is no need to additionally gain from any of the factors of $I_3$ and we simply bound all those, and their derivatives, by $n^\xi$ using Lemma  5. This results in a bound of $n^{\xi -(l-4)/2} e^{-l t/2} \prod _j \Vert \varDelta f^{(j)}\Vert _1$ for the terms of order $l$.

By combining the estimates on the terms of order three, four and higher order derivatives, and integrating in $t$ we obtain the bound (37). This completes the proof of Lemma 2. $\square$

## A. Extension of the local law

### Proof of Proposition 1

The statement for fixed $z,\eta$ follows directly from [4, Theorem 5.2], if $\eta \ge \eta _0:= n^{-3/4+\epsilon }$. For smaller $\eta _1$, using $\partial _\eta G(\text {i}\eta )=\text {i}G^2(\text {i}\eta )$, we write

59 $$\begin{aligned} \langle \varvec{x},[G(\text {i}\eta _1)-M(\text {i}\eta _1)]\varvec{y}\rangle&= \langle \varvec{x}, [G(\text {i}\eta _0)-M(\text {i}\eta _0)] \varvec{y}\rangle \nonumber \\&\quad +\text {i}\int _{\eta _0}^{\eta _1} \langle \varvec{x}, [G^2(\text {i}\eta )-M'(\text {i}\eta )] \varvec{y}\rangle {\text {d}}\!{}\eta \end{aligned}$$

and estimate the first term using the local law by $n^{-1/4+\xi }$. For the second term we bound

$$\begin{aligned} \left|\langle \varvec{x}, G^2 \varvec{y}\rangle \right|&\le \sqrt{ \langle \varvec{x},G^*G \varvec{x}\rangle \langle \varvec{y},G^*G \varvec{y}\rangle } = \frac{1}{\eta } \sqrt{\langle \varvec{x},\mathfrak {I}G \varvec{x}\rangle \langle \varvec{y},\mathfrak {I}G \varvec{y}\rangle }, \\ \left|\langle \varvec{x},M' \varvec{y}\rangle \right|&\lesssim \Vert \varvec{x}\Vert \Vert \varvec{y}\Vert \frac{1}{\eta ^{2/3}} \end{aligned}$$

from $\Vert M'\Vert \lesssim (\mathfrak {I}\widehat{m})^{-2}$ and (10), and use monotonicity of $\eta \mapsto \eta \langle \varvec{x},\mathfrak {I}G(\text {i}\eta )\varvec{x}\rangle$ in the form

$$\begin{aligned} \mathfrak {I}\langle \varvec{x}, G(\text {i}\eta )\varvec{x}\rangle \le \frac{\eta _0}{\eta } \langle \varvec{x}, \mathfrak {I}G(\text {i}\eta _0)\varvec{x}\rangle \prec \Vert \varvec{x}\Vert ^2 \Bigl (\frac{\eta _0^{4/3}}{\eta } + \frac{\eta _0^{2/3}}{\eta n^{1/2}} \Bigr ) \lesssim \Vert \varvec{x}\Vert ^2 \frac{n^{4\epsilon /3}}{n\eta }. \end{aligned}$$

After integration we thus obtain a bound of $\Vert \varvec{x}\Vert \Vert \varvec{y}\Vert n^{4\epsilon /3}/(n\eta _1)$ which proves the first bound in (13). The second, averaged, bound in (13) follows directly from the first one since below the scale $\eta \le n^{-3/4}$ there is no additional gain from the averaging, as compared to the isotropic bound.

In order to conclude the local law simultaneously in all $z,\eta$ we use a standard *grid argument*. To do so, we choose a regular grid of $z$’s and $\eta$’s at a distance of, say, $n^{-3}$ and use Lipschitz continuity (with Lipschitz constant $n^2$) of $(\eta ,z)\mapsto G^z(\text {i}\eta )$ and a union bound over the exceptional events at each grid point. $\square$

## Footnotes

1 The matrix $\varDelta ^\alpha$ is not to be confused with the Laplacian $\varDelta f$ in Girko’s formula (14).
